# The cost determinants of routine infant immunization services: a meta-regression analysis of six country studies

**DOI:** 10.1186/s12916-017-0942-1

**Published:** 2017-10-06

**Authors:** Nicolas A. Menzies, Christian Suharlim, Fangli Geng, Zachary J. Ward, Logan Brenzel, Stephen C. Resch

**Affiliations:** 1 Department of Global Health and Population, Harvard T.H. Chan School of Public Health, Boston, Massachusetts USA; 2 Center for Health Decision Science, Harvard T.H. Chan School of Public Health, Boston, Massachusetts USA; 3 Bill & Melinda Gates Foundation, Seattle, Washington USA

**Keywords:** Economic evaluation, Costs and cost analyses, Immunization programs, Vaccination

## Abstract

**Background:**

Evidence on immunization costs is a critical input for cost-effectiveness analysis and budgeting, and can describe variation in site-level efficiency. The Expanded Program on Immunization Costing and Financing (EPIC) Project represents the largest investigation of immunization delivery costs, collecting empirical data on routine infant immunization in Benin, Ghana, Honduras, Moldova, Uganda, and Zambia.

**Methods:**

We developed a pooled dataset from individual EPIC country studies (316 sites). We regressed log total costs against explanatory variables describing service volume, quality, access, other site characteristics, and income level. We used Bayesian hierarchical regression models to combine data from different countries and account for the multi-stage sample design. We calculated output elasticity as the percentage increase in outputs (service volume) for a 1% increase in inputs (total costs), averaged across the sample in each country, and reported first differences to describe the impact of other predictors. We estimated average and total cost curves for each country as a function of service volume.

**Results:**

Across countries, average costs per dose ranged from $2.75 to $13.63. Average costs per child receiving diphtheria, tetanus, and pertussis ranged from $27 to $139. Within countries costs per dose varied widely—on average, sites in the highest quintile were 440% more expensive than those in the lowest quintile. In each country, higher service volume was strongly associated with lower average costs. A doubling of service volume was associated with a 19% (95% interval, 4.0–32) reduction in costs per dose delivered, (range 13% to 32% across countries), and the largest 20% of sites in each country realized costs per dose that were on average 61% lower than those for the smallest 20% of sites, controlling for other factors. Other factors associated with higher costs included hospital status, provision of outreach services, share of effort to management, level of staff training/seniority, distance to vaccine collection, additional days open per week, greater vaccination schedule completion, and per capita gross domestic product.

**Conclusions:**

We identified multiple features of sites and their operating environment that were associated with differences in average unit costs, with service volume being the most influential. These findings can inform efforts to improve the efficiency of service delivery and better understand resource needs.

**Electronic supplementary material:**

The online version of this article (doi:10.1186/s12916-017-0942-1) contains supplementary material, which is available to authorized users.

## Background

Better evidence on immunization costs serves several goals—in the context of new vaccine adoption and service expansion, accurate cost estimates are critical inputs for cost-effectiveness and budget impact analyses of proposed policies. Understanding variation in costs across service outlets can also provide insight on site operations and suggest opportunities for improving efficiency. Despite these needs, there have been few empirical studies with sufficient sample size to provide precise cost estimates or describe inter-site cost variation, and studies have commonly used national-level budgeting data to investigate determinants and trends in immunization costs [1–3]. Prior empirical studies have found substantial variation both within [4, 5] and between countries [6], though some of these studies are now many years old.

The Expanded Program on Immunization Costing and Financing (EPIC) Project was designed to fill this knowledge gap, providing detailed data on routine immunization costs and financing in a large, representative sample of immunization sites in six countries (Benin, Ghana, Honduras, Moldova, Uganda, and Zambia) [7]. Information from these studies has already been used to improve information on unit costs [8–11], cost trends [12, 13], and financing [14, 15] within individual countries. We synthesized data from these country studies to create a unique pooled dataset of 316 sites to explore cross-country determinants of costs. We use these data to describe country- and site-level variation in routine immunization costs and identify systematic cost differences related to site operating characteristics. Given the observational nature of the data, the relationships we estimate are purely descriptive. However, the larger sample size allows us to provide a fine-grained description of how costs vary between similar sites, which in turn can suggest potential approaches for improving the efficiency of service delivery and allow a more precise understanding of resource needs.

## Methods

### Study sample

Countries were selected from a list of countries that had introduced pentavalent vaccine before 2011, and introduced pneumococcal or rotavirus vaccines in 2011 [7]. Countries varied by program performance (diphtheria, tetanus, and pertussis (DTP3) coverage from 75% to 93% [16]), income level (gross domestic product (GDP) per capita from US $531 to US $2277 [17]), and vaccination schedule. Inter-country differences are described in Additional file 1: Table S1. Sites were selected using a multi-stage cluster sample from a sampling frame of all public and non-governmental organization (NGO) facilities providing routine immunization. A total of 319 sites were enrolled. From each site, data were collected on costs, service volume, and site operating characteristics during January–December 2011, using a common approach [18]. Data were also collected on costs incurred at the subnational and national levels. Data collection occurred during 2012–2013.

### Data management and cleaning

We collated detailed data files from individual country studies and compiled them into a consistent format, with costs organized into standardized cost categories. We used automated tests to identify data anomalies, which were discussed and resolved with country teams. Three sites were excluded due to unresolved inconsistencies. Data cleaning and standardization resulted in minor differences with earlier studies’ country-level results [8–11]. Datasets and related materials are available at www.immunizationeconomics.org and archived on Dataverse [19].

### Total cost estimates

Total site-level economic costs were estimated from a program perspective using conventional methods, including all activities undertaken to provide routine immunization services to children aged 0–12 months. We focused on the 0–12 month old age group to allow greater standardization across the data collected through individual country-level studies. Site-level overheads were apportioned by direct allocation [18], and investments were amortized with a 3% discount rate [20]. Building space was costed as equivalent rental value or annualized construction cost, and donated items valued at market prices. Costs for equipment and other investments were based on replacement value. Costs for salaried labor were based on loaded salaries, and costs for non-salaried labor (“volunteers”) included volunteer stipends, per diems, and incentives. Additional file 1: Table S2 provides a full inventory of costs included in the study. Costs are reported in 2011 US dollars.

### Service delivery volume

Data on service delivery volume were extracted from routine reporting systems. We used the number of total immunization doses delivered to children aged 0–12 months by each site during the study period as the primary measure of service volume. We also report results using the number of children receiving their third immunization for DTP3 by each site during the study period as an alternate measure of service volume. DTP3 is a conventional proxy for the number of children completing the basic infant immunization schedule.

### Average cost estimates

For this study, we estimated the average cost per site, per dose, and per DTP3 for each country, and obtained confidence intervals with a multi-stage bootstrap with resampling at province and site levels. Average costs per dose and DTP3 were calculated as the sum of routine infant immunization costs for all sites in the sample divided by the sum of outputs (doses, or DTP3) for all sites in the sample. This value is equivalent to a weighted average of the site-level cost per outcome, with weights proportional to service volume. This represents an estimate of the cost per outcome assessed at the program level. We also report results as a simple average of the cost per outcome for all sites in the sample. Average cost estimates are reported with and without higher level program support (“above site costs”) included. All estimates were adjusted for survey weighting, with weights constructed as the inverse of the sampling probability for each site [18].

### Regression analyses

We explored site-level cost variation by regressing the log of total costs incurred at each site against several explanatory variables. We excluded higher level program support (above site costs) from this part of the analysis, as these costs are unlikely be explained by the site-level determinants considered as explanatory variables. We used a Bayesian hierarchical regression model to combine data from different country studies and account for the multi-stage sample design, with country- and province-level random effects. Using a hierarchical regression model allowed for sources of variation at the site, province, and country levels, and provided a framework for synthesizing data across countries. As we used a Bayesian approach, the uncertainty measures included in the Results section (such as 95% credible intervals provided around point estimates) represent posterior probabilities conditional on priors, likelihood, and regression model, unlike traditional confidence intervals. Regression models were estimated using an adaptive Hamiltonian Monte Carlo algorithm [21, 22]. Processing of data and results were undertaken in R [23]. Further details are provided in Additional file 1: Framework for regression analyses.

Explanatory variables included service volume (*log(doses)*), other site characteristics (government ownership (*Govt owned*), hospital status (*Hospital*) as an indicator of health system level, fraction of DTP3 delivered via outreach (*Fraction outreach*), fraction of resources devoted to management (*Fraction mgmt*), and the ratio of DTP3 to doses (*DTP3 per dose*) as a crude quality measure), and features of the operating environment (rural location (*Rural*), local antenatal care (ANC) coverage as a measure of healthcare access (*ANC4*), and wealth level in the local area relative to the national average (*Wealth ratio*)). We also included log per capita GDP in 2011(*log(GDP)*) as a crude index of inter-country price differences and other factors that vary with income level.

We fit a series of progressively more inclusive regression models including these explanatory variables. *Model 1* involves intercept plus country- and province-level random effects. *Model 2* incorporates Model 1 plus log(doses) and log(doses) squared. *Model 3* incorporates Model 2 plus log(GDP) and site characteristics. *Model 4* incorporates Model 3 plus features of the operating environment. *Model 5* incorporates Model 4 plus country-level random effects for log(doses).

Several variables were unavailable for some sites, and we investigated these in secondary analyses using the subset of sites for which data were available. As salary scales were fixed within each country, we used the site-level average salary, normalized to 1.0 for each country, as an index of staff training and seniority (*Staffing index,* not available for 10 sites). We also created an index for the average fraction of time staff spent working on immunization (*Dedication index,* not available for Uganda) to describe the extent to which staff were committed exclusively to immunization activities, as compared to being spread across multiple service areas. This was used as a measure of economies of scope for labor. Since the definition of a hospital may differ across countries, we used the number of inpatient beds, categorized into 0 beds, 1–9 beds, or 10+ beds, as an alternative indicator of health system level (*Inpatient beds*, not available for Moldova). Finally, we considered the number of days open per week (*Days per week*) and the distance to the vaccine collection point (*Distance*), both not available for Honduras. Table 1 provides summary information on all predictors. Additional file 1: Table S3 provides detailed variable definitions.

**Table 1:** Characteristics of sample

Outcome	Benin	Ghana	Honduras	Moldova	Uganda	Zambia
Sample size^a^	45	50	71	50	49	51
Total doses	7014 (4684)	3512 (3775)	4244 (7175)	557 (1172)	6561 (12144)	7069 (11343)
Total DTP3	665 (465)	378 (358)	280 (421)	54 (111)	682 (1401)	708 (1006)
Per capita GDP, 2011 USD	$745	$1594	$2277	$1971	$531	$1741
Rural	25/45	31/50	53/71	42/50	29/49	36/51
Government owned	41/45	47/50	71/71	50/50	37/49	49/51
Hospital	0/45	6/50	3/71	0/50	13/49	4/51
ANC4	0.69 (0.13)	0.83 (0.04)	0.90 (0.03)	0.93 (0.01)	0.52 (0.06)	0.62 (0.02)
Wealth ratio	1.17 (0.31)	1.03 (0.26)	1.09 (0.36)	0.92 (0.30)	1.10 (0.47)	1.42 (0.45)
Fraction outreach	0.20 (0.21)	0.63 (0.32)	0.14 (0.07)	0.00 (0.00)	0.38 (0.08)	0.46 (0.20)
Fraction management	0.02 (0.03)	0.07 (0.06)	0.05 (0.05)	0.19 (0.06)	0.13 (0.06)	0.12 (0.07)
Staffing index	1.00 (0.54)	1.00 (0.15)	1.00 (0.35)	1.00 (0.25)	1.00 (0.28)	1.00 (0.32)
DTP3 per dose	0.10 (0.03)	0.12 (0.06)	0.08 (0.01)	0.10 (0.02)	0.11 (0.02)	0.11 (0.02)
Days open per week	3.8 (1.5)	4.3 (1.9)	–	3.9 (1.6)	2.7 (2.2)	1.9 (1.5)
Distance to vaccine collection point (km)	15.5 (18.5)	8.0 (11.5)	–	19.6 (13.1)	12.9 (12.6)	50.2 (44.8)
Dedication index	0.50 (0.27)	0.45 (0.22)	0.34 (0.15)	0.26 (0.11)	–	0.32 (0.19)
Inpatient beds: 0	0/45	11/50	68/68	–	10/49	12/50
Inpatient beds: 1–9	12/45	34/50	0/68	–	17/49	22/50
Inpatient beds: 10+	33/45	5/50	0/68	–	22/49	16/50
Total catchment population (000 s)	20.7 (17.3)	14.1 (21.8)	–	5.2 (11.1)	41.4 (98.5)	22.2 (37.6)
DTP3 coverage^b^	0.82 (0.25)	0.75 (0.26)	0.85 (0.18)	0.88 (0.16)	0.62 (0.35)	0.81 (0.21)

^a^Sample size values represent the number of sites included in the main analysis for each country. All other values in table represent unweighted means for each county, and values in parentheses represent standard deviations

^b^Top-coded at 100%

We estimated a series of progressively more inclusive regression models, using the Watanabe-Akaike information criterion (WAIC) to describe model fit [24, 25]. Similar to AIC, WAIC is a statistic that measures the extent to which a model is able to explain variation in the data, while penalizing unnecessary model complexity. A lower WAIC implies a better fitting model. We calculated first differences using the regression results, describing the change in the cost per dose associated with a defined change in one or several predictors. We present equal-tailed 95% credible intervals to describe the uncertainty in these results, and we use the term “statistically discernable” to describe situations where these intervals exclude no effect. We generated graphs of total and average costs per dose as a function of service volume, using the Duan smearing estimator to retransform estimates to the absolute scale [26]. We calculated output elasticity as the percentage increase in outputs (service volume) for a 1% increase in inputs (total costs), averaged across the sample in each country. Using the fitted regression equations, we compared predicted unit costs (total costs divided by total doses) across the range of service delivery volume observed in each country, controlling for other factors.

### Robustness checks

We estimated several alternative regression specifications: (1) country-level fixed effects instead of random effects; (2) addition of district-level random effects; (3) a robust regression specification, with residuals assumed to follow a Student’s *t* distribution [27], to allow for outliers; and (4) adoption of non-informative priors (instead of weakly informative priors) for regression coefficients and variance terms. We also estimated results using the number of children receiving DTP3 as a measure of service volume in place of total doses.

We fit several regression models to investigate coverage as a predictor of site-level costs. We anticipated that unit costs of service delivery would be increasing at higher coverage levels, due to high marginal costs of reaching the very last members of the target population. However, the reported coverage measure (*DTP3 coverage*, reported as DTP3 divided by number of children < 1 years old in the catchment) exhibited substantial measurement error (many values > 100%), attributed to inaccurate population estimates. We omitted DTP3 coverage from the main analysis but undertook sensitivity analyses with (1) reported *DTP3 coverage* top-coded at 100% (i.e., revised to a value of 100% where the original value was > 100%), (2) an error-in-variables model for mismeasurement of *DTP3 coverage*, and (3) use of log catchment population (*log(Population)*) as an indirect approach for investigating the relationship between coverage and costs.

## Results

### Average cost estimates

In 2011 the study sites spent $6.79 million (2011 US dollars (USD)) providing routine immunization services to children < 1 year of age, recording 1.50 million vaccine doses and 141,000 DTP3. The distributions of site-level costs and service volume were strongly right skewed, with many small sites and few large sites. There was substantial variation in the cost per dose and per DTP3 in each country, with the coefficient of variation in the cost per dose ranging from 0.45 in Moldova to 1.37 in Ghana (mean 0.99 across the entire sample), such that the quintile of sites with the highest cost per dose were on average 440% more expensive than the quintile with the lowest cost per dose in each country. Additional file 1: Figure S1 shows distributions of the cost per site, per dose, and per DTP3 by country.

Table 2 reports average costs per site, per dose, and per DTP3 we estimated for each country. Average costs per site ranged from $4300 in Moldova to $27,900 in Zambia. Average costs per dose ranged from $2.75 in Benin to $13.63 in Moldova. Average costs per DTP3 ranged from $26.84 in Uganda to $139 in Moldova. Cost per outcome estimates calculated as a simple average across sites were 15–96% higher than values weighted by service volume, implying that sites with higher service volume had lower costs per outcome. Overheads for costs incurred above the site level represented an additional 6.0–22.5% on top of site-level costs.

**Table 2:** Average cost estimates, by country

Outcome	Benin	Ghana	Honduras	Moldova	Uganda	Zambia
Average cost estimates excluding above site-level costs						
Average cost per site (000 s)	$18.0 (15.1, 23.7)	$17.9 (14.1, 23.6)	$13.4 (10.5, 30.5)	$4.3 (2.3, 20.5)	$8.0 (6.2, 16.1)	$27.9 (19.6, 48.5)
Average cost per dose	$2.75 (2.50, 3.18)	$6.09 (4.11, 9.39)	$9.48 (7.08, 11.64)	$13.63 (10.94, 18.61)	$2.76 (1.94, 3.56)	$4.05 (2.86, 6.06)
Average cost per dose (simple average across sites)	$3.16 (2.83, 4.17)	$11.96 (5.11, 20.87)	$16.57 (10.95, 18.63)	$18.52 (15.14, 22.35)	$4.69 (3.04, 5.69)	$7.07 (6.00, 8.54)
Average cost per DTP3	$29.90 (27.07, 35.44)	$55.60 (39.97, 84.50)	$128 (100, 148)	$139 (111, 184)	$26.84 (18.96, 34.73)	$39.73 (30.52, 56.63)
Average cost per DTP3 (simple average across sites)	$35.18 (31.48, 46.59)	$106 (51.68, 180)	$223 (146, 262)	$210 (164, 253)	$40.12 (30.88, 45.25)	$64.91 (54.57, 82.12)
Mark-up for above site-level costs						
Subnational	4.5%	12.2%	8.8%	20.3%	12.3%	5.8%
National	1.5%	1.5%	9.9%	2.2%	7.7%	1.5%
Total	6.0%	13.7%	18.7%	22.5%	20.0%	7.4%
Average cost estimates including above site-level costs						
Average cost per site (000 s)	$19.0 (16.0, 25.1)	$18.9 (16.1, 26.9)	$14.2 (12.5, 36.2)	$4.5 (2.9, 25.1)	$8.5 (7.4, 19.3)	$29.5 (21.1, 52.1)
Average cost per dose	$2.91 (2.65, 3.37)	$6.45 (4.67, 10.67)	$10.05 (8.41, 13.82)	$14.44 (13.41, 22.79)	$2.93 (2.32, 4.27)	$4.29 (3.07, 6.51)
Average cost per DTP3	$31.68 (28.69, 37.55)	$58.91 (45.44, 96.07)	$135 (119, 176)	$147 (136, 225)	$28.44 (22.74, 41.66)	$42.09 (32.78, 60.82)

Point estimates represented mean estimates adjusted for survey weighting. Values in brackets represent 95% confidence intervals estimated via multi-stage bootstrap with 50,000 replicates, adjusted for survey weighting. Main cost per outcome estimates (rows 2 and 4) represent total costs for sites in the sample divided by total service volume. Outcomes in rows 3 and 5 (simple average across sites) represent a simple average of the cost per dose (or DTP3) across sites in the sample. Above-site program support costs applied as a fixed mark-up on site-level costs

### Cost determinants

Table 3 reports a series of progressively more inclusive regression models fit to log total costs.

**Table 3:** Results for regressions of log total cost on service volume and other potential predictors

Variable^a^	Model specification				
	1	2	3	4	5
Intercept	9.44 (0.45)	9.44 (0.25)	9.43 (0.16)	9.43 (0.16)	9.48 (0.13)
Service volume					
log(doses)	–	1.10 (0.03)	1.10 (0.03)	1.13 (0.04)	1.04 (0.20)
log(doses) squared	–	–0.04 (0.02)	–0.01 (0.02)	–0.01 (0.02)	0.10 (0.03)
Other predictors					
log(GDP)	–	–	0.41 (0.16)	0.31 (0.17)	0.39 (0.14)
Government owned	–	–	–0.13 (0.09)	–0.14 (0.09)	–0.15 (0.08)
Hospital	–	–	0.34 (0.09)	0.31 (0.09)	0.27 (0.08)
Percent outreach	–	–	0.11 (0.04)	0.11 (0.04)	0.08 (0.03)
Percent management	–	–	0.13 (0.03)	0.13 (0.03)	0.13 (0.03)
DTP3 per dose	–	–	0.15 (0.02)	0.15 (0.02)	0.13 (0.02)
Rural	–	–	–	–0.02 (0.06)	–0.04 (0.06)
ANC4	–	–	–	0.14 (0.08)	0.12 (0.07)
Wealth ratio	–	–	–	–0.06 (0.04)	–0.07 (0.03)
Random effects included					
Country r.e.s for intercept	+	+	+	+	+
Province r.e.s for intercept	+	+	+	+	+
Country r.e.s for log(doses)	–	–	–	–	+
Variance parameters					
Error term	0.86 (0.04)	0.39 (0.02)	0.35 (0.02)	0.35 (0.02)	0.32 (0.01)
SD of country r.e.s, intercept	0.93 (0.48)	0.55 (0.27)	0.34 (0.24)	0.32 (0.23)	0.24 (0.21)
SD of province r.e.s, intercept	0.44 (0.10)	0.19 (0.04)	0.19 (0.04)	0.20 (0.04)	0.14 (0.03)
SD of country r.e.s, log(doses)	–	–	–	–	0.46 (0.23)
WAIC^b^	828.2	335.2	273.2	270.9	212.4
Sample size	316	316	316	316	316

^a^Country and province random effects not shown. Predictors are standardized; thus, fitted coefficients for continuous variables (e.g., log(doses)) represent the increase in log total costs observed for a 1.0 standard deviation increase in the variable. Values in parentheses represent standard errors

^b^Watanabe-Akaike information criterion (*WAIC*) describes out-of-sample prediction accuracy for the fitted model, with lower values suggesting better model fit

*SD* standard deviation, *r.e.* random effect

Results show a strong relationship between service volume and total costs, evident in the substantial reduction in WAIC produced by including service volume in the regression model. Including variables for site characteristics and features of the operating environment produced additional reductions in WAIC (though insignificant in the latter case). The additional reduction in WAIC associated with inclusion of random effects for log(doses) (Model 5 vs. Model 4) indicates statistically discernable inter-country variation in the relationship between total costs and service delivery volume.

As the regressions used log-transformed costs and standardized variables, the regression coefficients are difficult to interpret directly [28]. First differences were calculated to demonstrate the implications of the regression results (Table 4), based on the best fitting regression model (Model 5). These results describe the percentage difference in the cost per dose produced by change in an individual predictor or subset of predictors. As the regression model included service volume, the first differences calculated for other predictors apply to both the cost per dose and total site-level costs, for sites of equal service volume. For continuous predictors, first differences were calculated to represent the difference between the 25% and 75% percentiles of each predictor.

**Table 4:** First differences calculated from regression results

Comparison^a^	Percentage difference in average cost per dose^b^
Each country, as compared to the overall mean (includes differences in per-capita GDP, controls for other predictors):	
*Uganda (per capita* GDP 2011 = US$ 531)	-55% (-65, -42)
*Benin (per capita* GDP 2011 = US$ 745)	–27% (-44, -8.2)
*Ghana (per capita* GDP 2011 = US$ 1594)	–9.6% (–26, 8.1)
*Zambia (per capita* GDP 2011 = US$ 1740)	22% (1.4, 47)
*Moldova (per capita* GDP 2011 = US$ 1971)	27% (2.8, 53)
*Honduras (per capita* GDP 2011 = US$ 2277)	43% (21, 65)
Per capita GDP doubled	68% (16, 133)
Government-owned sites, as compared to non-government-owned sites	–14% (–27, –0.8)
Hospital-based sites, as compared to other sites	32% (12, 55)
Percentage of doses delivered via outreach 32% points higher^c^	9.8 (1.6, 18)
Share of program activity to management 12% points higher^c^	23% (12, 33)
DTP3 as percent of all doses 1.5% points higher^c^	13% (8.4, 18)
Rural sites, as compared to urban and per-urban sites	–3.6% (–14, 7.5)
ANC4 coverage 30% points higher^c^	25% (–2.5, 61)
Wealth ratio 52% points higher^c^	–8.8% (–16, –0.8)
Service delivery volume (doses) doubled, as compared to a site with the median no. doses for each country:	
*Uganda*	-14% (-19, -7.7)
*Benin*	-16% (-23, -7.9)
*Ghana*	-32% (-37, -27)
*Zambia*	-27% (-32, -22)
*Moldova*	-13% (-17, -8.9)
*Honduras*	-18% (-21, -14)
*Overall*	-19% (-32, -4.0)

^a^Values calculated from the results of regression Model 5, controlling for all other model parameters except for those described in the comparison

^b^Calculated as one minus the average cost per dose for the given scenario divided by average cost per dose in comparator scenario (thus, “–50%” would indicate a halving of costs and “50%” would indicate a 50% increase in costs). Values represent posterior means, and values in parentheses represent equal-tailed 95% credible intervals

^c^Magnitude of change equal to the difference between the 25th and the 75th percentile of the sample distribution for each variable

**Fig. 1:**
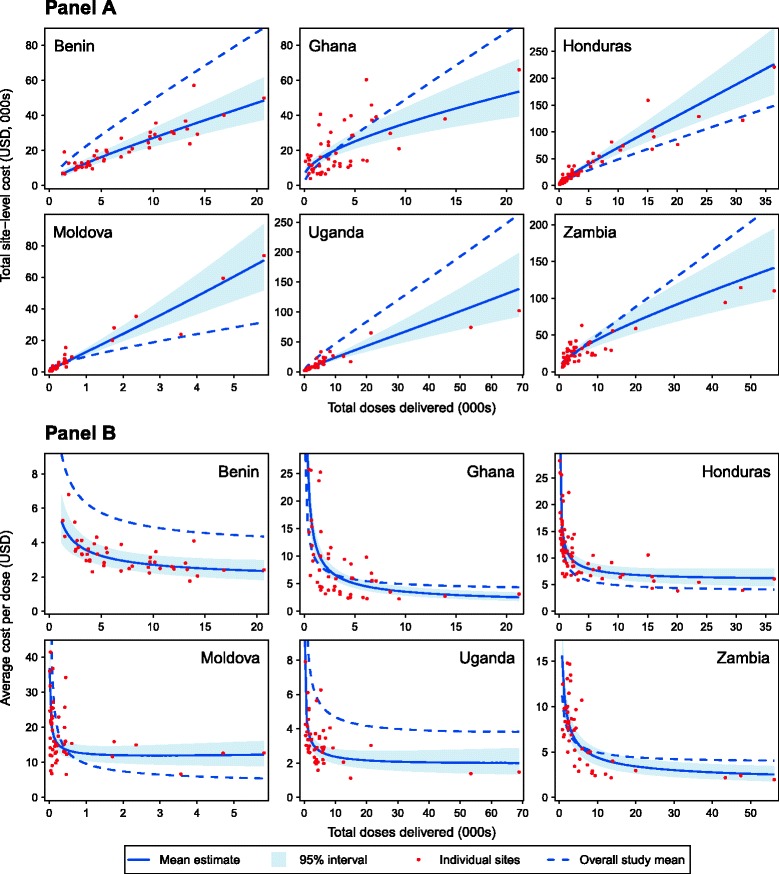
Total site-level costs (Panel **A**) and cost per dose (Panel **B**) as a function of service volume (reported doses). Mean line and 95% credible intervals calculated from the results of Model 5

Figure 1 presents total and average costs for each country as a function of service volume, based on Model 5. Additional file 1: Figure S2 presents these relationships plotted on a log scale to allow better visualization of model fit. The average cost curves reveal differences between countries, though all curves reflect a convex, monotonically declining relationship between average costs and service volume. These results demonstrate a strong negative relationship between service volume and average costs in each country, and we estimated output elasticity values of 1.39 (1.16–1.68), 2.52 (1.97–3.31), 1.41 (1.31–1.54), 1.27 (1.17–1.40), 1.34 (1.19–1.52), and 1.87 (1.56–2.28) in Benin, Ghana, Honduras, Moldova, Uganda, and Zambia respectively. This implies that, across the entire sample, a site with 10% higher costs would have on average 14% (12–17) higher service volume, controlling for other effects. As a consequence, the largest 20% of sites (by number of doses) were estimated to have a cost per dose that was 55% (40–67), 41% (24–55), 83% (77–88), 68% (59–76), 52% (38–63), and 67% (59–75) percent lower than that for the smallest 20% of sites in Benin, Ghana, Honduras, Moldova, Uganda, and Zambia respectively, controlling for other factors.

### Additional predictors

Several predictors (staffing index, immunization days per week, distance to vaccine delivery, dedication index, and number of inpatient beds) were not available for the entire sample. We estimated regression models for total site-level costs using the subset of sites with data available on these predictors, and in each case identified a statistically discernable positive relationship with costs (Models 6–10, Additional file 1: Table S4). Coefficients for other predictors showed some variation across these specifications, likely due to the different samples to which models were fit. First differences calculated from these results showed a 5.9% (95% credible interval 2.4–9.5) cost increase associated with an 86 percentage point increase in the staffing index (missing for 10 sites). There was a 4.2% (1.5–7.0) cost increase with each doubling in distance to the vaccine collection point, and a 10.0% (2.8–18.0) cost increase with each additional day open per week (both variables not reported for Honduras). A 25 percentage point increase in the dedication index (extent to which staff were dedicated exclusively to providing immunization services) was associated with a 21% (15–28) cost increase (excludes Uganda). Finally, when the number of inpatient beds was used as an indicator for health system level (excluding Moldova), sites with 0–9 beds were 4.7% (–9.6 to 19) more expensive than sites without beds, and sites with 10+ inpatient beds were 21% (2.3–42) more expensive, consistent with the results for hospital status reported in Table 4.

### Alternative regression specifications

Using country fixed effects instead of random effects did not affect results, with model fit statistics, coefficients, and standard errors universally similar (Additional file 1: Table S5), and a Hausman test failed to reject the random effects specification. Similarly, including district-level random effects (Additional file 1: Table S6), adopting a robust regression approach (Additional file 1: Table S7), or adopting non-informative priors (Additional file 1: Table S8) had little impact on regression results. Additional file 1: Table S9 shows results for regressions using log(DTP3) as a measure of service volume. This produced little change in most coefficients, except for a minor increase in the strength of the relationship estimated for hospital status, and reversal of the sign of the coefficient for DTP3 per dose. Additional file 1: Figure S3 shows total and average cost curves plotted for DTP3.

Additional file 1: Table S10 presents various approaches for including DTP3 coverage in regression equations. When a top-coded estimate of DTP3 coverage was included, this produced a statistically discernable negative relationship with total costs. Results were similar when an error-in-variables model was used for DTP3 coverage, although no longer statistically discernable. Including log catchment population in the regression equations produced a statistically discernable positive relationship. This is consistent with the two results for DTP3 coverage, as coverage will be negatively related to total catchment population, conditional on service volume.

## Discussion

Costs per dose ranged from $2.75 (2011 USD) in Benin and Uganda to $13.63 in Moldova. Costs per DTP3 ranged from $27 in Uganda to $139 in Moldova. These values are substantially greater than those reported by earlier studies [4–6, 29], reflecting both higher price levels and expanded vaccine schedules. Inter-country differences in this study are likely due to similar factors, with countries with higher costs per DTP3—Moldova and Honduras—experiencing comparatively higher income levels and longer vaccine schedules. Some of our estimates differ from earlier estimates from the EPIC country studies [7], reflecting refinements to cost and outcomes data during data cleaning. Different approaches to calculating the average cost per outcome (Table 2) also contributed to variation in estimates, due to the strong relationship between service volume and the site-level cost per outcome.

Regression analyses revealed several predictors with a statistically discernable relationship with costs. In each country, higher service volume was strongly associated with lower average costs, with output elasticity ranging from 1.27 to 2.52 across the six countries, with a mean value of 1.63, and the largest 20% of sites in each country had a cost per dose that was on average 61% lower than that observed for the smallest 20% of sites. For most countries there were many small sites with high average costs, and these small sites exhibited substantial variation in unit costs. The reduction in unit costs associated with higher service volume was only minimal for sites at the upper end of the service volume distribution, and these large sites exhibited only minor variation in unit costs. While this suggests that greater reductions in average costs might be achieved through efforts to improve efficiency in small sites, it is not clear that these sites should be prioritized, as large total cost reductions might be possible with only small reductions in unit costs at large sites.

Higher health system level (proxied by hospital status) was associated with higher costs, possibly reflecting differences in available staffing and infrastructure. Outreach-based delivery was also associated with higher costs, possibly due to the additional resources required to deliver mobile services while maintaining infrastructure for facility-based provision. We designed the staffing index to test whether additional productivity of more highly trained and senior staff would counterbalance their higher salaries, yet the positive coefficient on this variable suggests otherwise. Greater distance to vaccine collection and more days open per week (implying lower daily service volume) were both associated with higher costs. A higher fraction of costs for management and greater completion of the vaccination schedule (proxied by the DTP3:doses ratio) were both found to have a strong positive relationship with costs. The positive coefficient on the dedication index implied lower cost for sites where staff are spread across multiple services (rather than being fully committed to immunization services), consistent with economies of scope and a greater ability to adjust staffing with variable demand. Some of these relationships were stronger than others, but they were generally robust to changes in regression specification. After controlling for site characteristics, there was little difference between rural and non-rural sites, despite rural sites having substantially higher costs per dose in crude comparisons. Per capita GDP was found to have a positive relationship with costs per dose. This likely reflects differences in price levels between countries, but it could also be related to the many other factors that vary with country income level. As only six countries were represented in this sample, we were unable to decompose the effect of these country-level factors.

The negative coefficient estimated for coverage was unexpected. We expected costs to increase with coverage, reasoning that improving coverage would require attracting progressively harder-to-reach clients, driving up marginal costs. It is possible that for a given service volume, sites with lower coverage have more widely dispersed or immunization resistant catchment populations, leading to higher average costs. Alternately, if staffing and other immunization resources were determined by catchment population size, lower attendance could produce both lower coverage and higher costs per outcome. While mismeasurement of reported coverage dictates that no firm conclusions should be drawn, the importance of coverage for immunization program strategy suggests that further investigation would be valuable.

Several relationships described above have been investigated in country-level studies [12, 13], with generally consistent results. While we have described possible mechanisms for these findings, the ability of our study design to identify causal relationships is weak. Many of the findings are consistent with multiple explanations (including omitted variable bias) and should be viewed as hypothesis generating rather than confirming any specific causal relationship. For this reason, the total and average cost curves we estimate for each country (Fig. 1) demonstrate how costs vary between sites of different size, but additional (and potentially unwarranted) assumptions are required to interpret them as the cost curves that would be experienced by sites attempting to increase service volume. Other limitations include the exclusion of predictors that could not be calculated for a sufficient number of sites to be considered in the analysis (for example, vaccine stock-outs and wastage rates) or that primarily varied at the country level (for example, input prices and vaccine schedules), and for which we had minimal power to investigate. While we attempted to create explanatory variables with consistent interpretations, it is likely that some of these constructs varied between countries, such as the definition of outreach services, or hospital status. Finally, the results for Ghana showed noticeably greater unexplained variation compared to the other countries (Fig. 1, Additional file 1: Figure S2), yet the reasons for this are unclear.

## Conclusions

The EPIC Project is the largest study to systematically investigate routine immunization costs. Prior studies of immunization costs have been hampered by small sample sizes, producing noisy average cost estimates and providing little systematic evidence on the variation in costs within countries. By enrolling a large number of immunization sites and using standardized data collection approaches across countries, the EPIC Project provides a unique opportunity to investigate and understand variation in the costs of immunization service delivery as well as generate more precise national cost estimates. This analysis examined many features of sites and their operating environments, but further investigation of these data is possible. The combined EPIC data are freely available for use by other investigators [19], with the hope that open access will maximize the utility of these data for understanding the costs of routine immunization services. The major findings of this study have relevance for policy makers at country and global levels. The substantial variation within and between countries suggests it is challenging now to use a single number to represent average immunization costs. This variation can also lead to an open dialogue about ways to reduce variation and improve value for money in immunization services.

## Additional file

Additional file 1: Supplementary material for “The cost determinants of routine infant immunization services: a meta-regression analysis of six country studies”. (DOCX 426 kb)

## Abbreviations

DTP3: Third immunization for diphtheria, tetanus, and pertussis
EPIC: Expanded Program on Immunization Costing and Financing
GDP: Gross domestic product
NGO: Non-governmental organization
WAIC: Watanabe-Akaike information criterion
