# Supplementary material for: The cost determinants of routine infant immunization services: a meta-regression analysis of six country studies
Source: BMC Med. 2017 Oct 6;15:178. doi: 10.1186/s12916-017-0942-1 (PMC5629762; doi:10.1186/s12916-017-0942-1)
Supplement: Supplementary file 1 — Supplementary material for “The cost determinants of routine infant immunization services: a meta-regression analysis of six country studies”. (DOCX 426 kb) [file 12916_2017_942_MOESM1_ESM.docx]

Supplementary material for “The cost determinants of routine infant immunization services: a meta-regression analysis of six country studies”.

**CONTENTS**

**Table S1:** Characteristics of countries included in the study.

**Table S2:** Cost **categories** included in study.

**Table S3: Variable definitions.**

Figure S1: **Sample distribution of cost per site, per dose, and per DTP3 for each country.**

**Framework for regression analyses.**

Figure S2: Total and average cost curves for total doses, calculated from the results of Model 5 (log-scale).

**Table S4: Results for regressions using additional predictors with incomplete reporting.**

**Table S5: Sensitivity analysis for regression of log total cost on doses and other predictors: country-level fixed effects in place of country-level random effects.**

**Table S6: Sensitivity analysis for regression of log total cost on doses and other predictors: inclusion of district-level random effects.**

**Table S7: Sensitivity analysis for regression of log total cost on doses and other predictors: robust regression with errors distributed Student *t*.**

**Table S8: Sensitivity analysis for regression of log total cost on doses and other predictors: non-informative priors used for regression coefficients and variance terms.**

**Table S9: Results for regressions using total DTP3 as measure of service delivery volume.**

Figure S3: Total and average cost curves for total DTP3, calculated from the results of model 15.

**Table S10: Sensitivity analysis for regression of log total cost on doses and other predictors: different formulations of DTP3 coverage.**

Measurement error model for DTP3 coverage (Model 22).

**Figure S4: Histogram of reported DTP3 coverage.**

**Table S11: National DTP3 coverage estimates and values estimated by measurement error model (Model 22).**

**Figure S5: Reported vs. estimated values of DTP3 coverage from measurement error model (Model 22).**

**TABLE S1:** CHARACTERISTICS OF COUNTRIES INCLUDED IN THE STUDY

| Country | GDP per capita (USD, 2011) [1] | DTP3 coverage (2011) [2] | Full vaccination schedule* (2011) [3] |
| --- | --- | --- | --- |
| Benin | $745 | 75% | BCG (birth), DTwPHibHep (6, 10, 14 weeks), Measles  (9 months), OPV (birth; 6, 10, 14 weeks), Pneumo_conj (6, 10, 14 weeks), TT (1st contact pregnancy; +1, +6 months; +1, +1 year ), Yellow Fever (9 months) |
| Ghana | $1594 | 91% | BCG (birth), DTwPHibHep (6, 10, 14 weeks), Measles  (9 months), OPV (6, 10, 14 weeks), TT (1st contact pregnancy; +1, +6 months; +1, +1 year ), Yellow Fever (9 months) |
| Honduras | $2277 | 91% | BCG (birth), DT (2, 4, 6, 18 months; 4 years; high-risk groups), DTwP (18 months; 4 years) DTwPHibHep (2, 4, 6 months), HepB (birth; 1st contact; +1 +6 months; high-risk groups), Influenza (6-35 months (2 doses); >60 years; high-risk groups), IPV (2, 4, 6, 18 months; high-risk groups), MMR (12 months), MR (>50 years; high-risk groups), OPV (2, 4, 6, 18 months), Pneumo_conj (2, 4, 6 months; 1 year), Rotavirus (2, 4 months), Td (11 years, pregnancy), Yellow fever (>1 year) |
| Moldova | $1971 | 93% | BCG (2 days; 7 years), DT (7 years), DTwP (22 months), DTwPHibHep (2, 4, 6 months), HepB (birth), MMR (12 months; 7, 15 years), OPV (2, 4, 6, 22 months; 7, 15 years), Pneumo_conj (subnational), Rotavirus (subnational) |
| Uganda | $531 | 82% | BCG (birth), DTwPHibHep (6, 10, 14 weeks), HPV (10-12 years; +4 weeks; +5 months (subnational)), Measles  (9 months), OPV (birth; 6, 10, 14 weeks), TT (childbearing-aged women (15-49 years); +1, +6 months; +1, +1 year) |
| Zambia | $1741 | 81% | BCG (birth), DTwPHibHep (6, 10, 14 weeks), Measles  (9 months), OPV (birth; 6, 10, 14 weeks), TT (first contact pregnancy; +1, +1, +6 months; +1, +1 year) |

* The analysis considered the subset of these schedules delivered to children 0-12 months of age.**TABLE S2:** COST **CATEGORIES** INCLUDED IN STUDY

| Cost category | Details |
| --- | --- |
| Labor | Shared and immunization-specific personnel salary, plus volunteer time |
| Vaccine | Vaccines and vaccine supplies, including wastage |
| Other supplies | Cost of stationery, IEC materials, and other supplies for the immunization program |
| Transport | Vehicles, vehicle maintenance, fuel, and other transportation-related costs |
| Cold chain | Cold chain equipment, maintenance and energy consumed |
| Infrastructure | Buildings, operating cost, utilities and all other cost, including printing, other equipment, other recurrent |
| Per diem | Any allowances paid to paid or volunteer workers for immunization-related activities |

**TABLE S3: VARIABLE DEFINITIONS**

| Variable | Definition |
| --- | --- |
| Total costs | Total economic costs of routine immunization services provided to children 0-12 months old during January-December 2011, estimated from a provider perspective. For regression analyses these cost estimates excluded higher-level program support (‘above site-level costs’). Valued in 2011 US dollars. Log-transformed value used in regression model. |
| Total doses | Reported number of immunization doses delivered to children 0-12 years old during January-December 2011. Log-transformed value used in regression models. |
| Total DTP3 | Reported number of DTP3 delivered during January-December 2011, including all possible formulations. Log-transformed value used in regression models. |
| Per capita GDP | Per capita GDP for each country in 2011[1], values in 2011 USD (country level variable). |
| Rural | Indicator variable for rural location of facility (excludes ‘compound’, ‘peri-urban’ and ‘semi-urban’ classifications). Variable centered to mean zero prior to analysis. |
| Govt owned | Indicator variable for government ownership of facility (excludes ‘CHAG/NGOs’, ‘NGO/Religious’ and ‘Private’ classifications). Variable centered to mean zero prior to analysis. |
| Hospital | Indicator variable for hospital status of facility (includes all facilities classified as hospitals plus health center IV in Uganda). Variable centered to mean zero prior to analysis. |
| ANC4 | Estimate for coverage of the 4^th^ ANC visit in the local area. Values obtained by reanalysis of national population survey data (DHS), with spatial interpolation. |
| Wealth ratio | Estimate for wealth level in the local area relative to the national average. Values obtained by reanalysis of national population survey data (DHS), with spatial interpolation. |
| Percent outreach | One minus the fraction of immunization doses reported as being delivered in the facility, reported as a percentage. Variable standardized (scaled to mean zero and unit standard deviation) prior to analysis. |
| Percent mgmt | Percentage of reported costs devoted to management (a standard classification of programmatic activities applied in each country). |
| Staffing index | Total salaries costed for immunization services divided by total FTE for immunization, divided by the sample average for each country. |
| DTP3 per dose | Ratio of reported DTP3 to reported doses provided to children 0-12 months old. For a given country, higher values will indicate greater completion of the vaccination schedule. |
| Days open per week | The reported number of days per week during which immunization services are offered. |
| Distance to vaccine collection point | The reported distance from the site to the vaccine collection point in km. |
| Dedication index | sum(FTE_i^2)/sum(FTE_i), where FTE_i is a vector of the FTE that each staff member devotes to immunization. This calculation provides the average fraction of time that staff spend working on immunization (for those staff involved with providing immunization services). |
| Inpatient beds: 0 | Indicator variable for whether the site has 0 inpatient beds, as an alternative indicator of health system level. |
| Inpatient beds: 1-9 | Indicator variable for whether the site has 1-9 inpatient beds, as an alternative indicator of health system level. |
| Inpatient beds: 10+ | Indicator variable for whether the site has ≥10 inpatient beds, as an alternative indicator of health system level. |
| Total catchment population | The total population of the catchment area of the facility reported for 2011, in 1000s. |
| DTP3 coverage | Reported DTP3 coverage, equivalent to total DTP3 divided by reported number of children 0-122 months old in the catchment area. |

**FIGURE S1: SAMPLE DISTRIBUTION OF COST PER SITE, PER DOSE, AND PER DTP3 FOR EACH COUNTRY*.**

* Distributions adjusted for survey weighting.

**FRAMEWORK FOR REGRESSION ANALYSES**

Regression analyses were undertaken using a multilevel model to account for the multi-stage sample design, with clustering at country- and province-level. The general structure of this regression equation is shown below, whereby the natural log of total costs for facility *i* ($ln\left( {TC}_{i} \right)$) was assumed equal to a linear combination of predictors ($\beta_{0}+\sum_{j} \beta_{j}X_{ij}$), plus random effects for country ($\delta_{c_{i}}$) and province ($\delta_{p_{i}}$) as well as an error term ($\varepsilon_{i}$). By adopting log costs as the dependent variable, this specification assumes a multiplicative relationship between individual predictors and total cost.

$$ln\left( {TC}_{i} \right)= \beta_{0}+\sum_{j} \beta_{j}X_{ij}+ \delta_{c_{i}}+\delta_{p_{i}}+\varepsilon_{i}$$

Regression models were operationalized within a Bayesian framework, with weakly-informative prior distributions specified for all model parameters. Normal priors were assumed for all regression coefficients. Random effect and error terms were assumed to follow a Normal distribution centered at zero with a standard deviation estimated from the data. Half-Cauchy hyper-priors were used for the standard deviation of the random effect and error terms [4]. In sensitivity analysis we assessed the impact of assuming non-informative priors (improper Uniform distributions) for regression coefficients and variance terms.

$$\beta_{j}\sim Norm\left( 0 , 10 \right) for j in \epsilon[0.. J]$$

$$\delta_{c}\sim Norm\left( 0 , \sigma_{C}^{2} \right) for c \epsilon[1.. 6]$$

$$\delta_{p}\sim Norm\left( 0 , \sigma_{P}^{2} \right) for p \epsilon[1.. 36]$$

$$\varepsilon\sim Norm\left( 0 , \sigma^{2} \right)$$

$$\sigma_{C}\sim HalfCauchy\left( 0 , 5 \right)$$

$$\sigma_{P}\sim HalfCauchy\left( 0 , 5 \right)$$

$$\sigma\sim HalfCauchy\left( 0 , 5 \right)$$

All predictors were standardized before fitting regression equations, with indicator variables centered at mean zero and other variables scaled to mean zero and unit standard deviation. Survey weights were not used when estimating regression models, but used to calculate the covariate distribution for each country when making predictions from the fitted model [5]. We used the Watanabe-Akaike information criterion (WAIC), which approximates out-of-sample prediction accuracy for the fitted model, as a measure of model fit [6]. We used an operationalization of WAIC described by Vehtari and Gelman [7].

Regression models were estimated using an adaptive Hamiltonian Monte Carlo algorithm [8] as implemented by the Stan software package, version 2.16.2 [9, 10]. For each regression model we ran 4 chains for 5000 iterations (2500 warm-up, 2500 sampling), obtaining 10000 posterior draws used for subsequent analyses. For the models shown in the main analysis (Models 1 to 5 in Table 3), $\hat{R}$ values were all equal to 1.0 ($\hat{R}$values above 1.0 indicate poor chain convergence), there were zero divergences (divergences are an indicator of poor sampling), and the minimum effective sample size for model parameters was 1329, and typical values were >2000 (low values would indicate imprecision in parameter estimates due to Monte Carlo error). We have added this reference to the text. Processing of data and results were undertaken in R version 3.3.3 [11].

FIGURE S2: TOTAL AND AVERAGE COST CURVES FOR TOTAL DOSES, CALCULATED FROM THE RESULTS OF MODEL 5 (LOG-SCALE).

**TABLE S4: RESULTS FOR REGRESSIONS USING ADDITIONAL PREDICTORS WITH INCOMPLETE REPORTING.**

| Variable* |  | Model specification* | | | |  | |
| --- | --- | --- | --- | --- | --- | --- | --- |
|  | 6 | | 7 | 8 | 9 | | 10 |
| Intercept | 9.30 (0.57) | | 9.26 (0.82) | 9.22 (0.87) | 9.25 (0.99) | | 9.63 (0.38) |
| *Service volume* |  | |  |  |  | |  |
| log(doses) | 0.73 (0.14) | | 0.71 (0.13) | 0.69 (0.16) | 0.70 (0.15) | | 0.66 (0.13) |
| log(doses) sq | 0.06 (0.01) | | 0.06 (0.01) | 0.06 (0.02) | 0.06 (0.02) | | 0.06 (0.01) |
| *Other predictors* |  | |  |  |  | |  |
| log(GDP) | -0.08 (0.61) | | -0.16 (0.83) | -0.16 (0.17) | -0.27 (0.83) | | 0.18 (0.39) |
| Govt owned | -0.21 (0.08) | | -0.13 (0.08) | -0.15 (0.09) | -0.17 (0.12) | | -0.15 (0.08) |
| Hospital | 0.27 (0.08) | | 0.36 (0.09) | 0.35 (0.09) | 0.08 (0.11) | | --- |
| Percent outreach | 0.05 (0.02) | | 0.07 (0.02) | 0.07 (0.04) | 0.05 (0.02) | | 0.05 (0.02) |
| Percent mgmt | 0.09 (0.02) | | 0.08 (0.02) | 0.08 (0.03) | 0.07 (0.02) | | 0.07 (0.02) |
| DTP3 per dose | 0.09 (0.02) | | 0.13 (0.02) | 0.14 (0.07) | 0.08 (0.02) | | 0.10 (0.02) |
| Rural | -0.01 (0.06) | | -0.06 (0.06) | 0.02 (0.10) | 0.05 (0.07) | | -0.07 (0.06) |
| ANC4 | 0.04 (0.03) | | 0.06 (0.03) | 0.04 (0.06) | 0.02 (0.03) | | 0.04 (0.03) |
| Wealth ratio | -0.06 (0.03) | | -0.08 (0.04) | -0.08 (0.05) | -0.06 (0.03) | | -0.09 (0.03) |
| Staffing index | 0.07 (0.02) | | --- | --- | --- | | --- |
| log(Distance) | --- | | 0.08 (0.03) | --- | --- | | --- |
| Days per week | --- | | --- | 0.07 (0.03) | --- | | --- |
| Dedication index | --- | | --- | --- | 0.16 (0.02) | | --- |
| Inpatient beds: 0-9 | --- | | --- | --- | --- | | 0.04 (0.07) |
| Inpatient beds: 10+ | --- | | --- | --- | --- | | 0.19 (0.08) |
| *Random effects included* |  | |  |  |  | |  |
| Country r.e.s for intercept | + | | + | + | + | | + |
| province r.e.s for intercept | + | | + | + | + | | + |
| Country r.e.s for log(doses) | + | | + | + | + | | + |
| *Variance parameters* |  | |  |  |  | |  |
| Error term | 0.31 (0.01) | | 0.32 (0.02) | 0.32 (0.02) | 0.30 (0.01) | | 0.32 (0.01) |
| SD of country r.e.s, intercept | 1.27 (0.70) | | 1.62 (1.03) | 1.66 (1.20) | 1.65 (1.14) | | 0.64 (0.57) |
| SD of province r.e.s, intercept | 0.13 (0.03) | | 0.14 (0.04) | 0.16 (0.04) | 0.16 (0.04) | | 0.13 (0.04) |
| SD of country r.e.s, log(doses) | 0.28 (0.15) | | 0.26 (0.17) | 0.31 (0.20) | 0.28 (0.18) | | 0.25 (0.16) |
| WAIC** | 196.9 | | 167.0 | 167.8 | 151.2 | | 176.4 |
| Sample size | 307 | | 242  excl. Honduras | 245  excl. Honduras | 258,  excl. Uganda | | 262  excl. Moldova |

*** Models 6-9 is similar to Model 4 except with the addition of staffing index, log distance to vaccine collection point, number of immunization days per week, and the dedication index, respectively. Model 10 is similar to Model 4 except with hospital status replaced by indicators for 0-9 and 10+ inpatient beds as a measure of health system level.**

**** Fit measures for these models are not comparable between models (or to values shown in Table 3) as they were fit to different subsets of the dataset.**

**TABLE S5: SENSITIVITY ANALYSIS FOR REGRESSION OF LOG TOTAL COST ON DOSES AND OTHER PREDICTORS: COUNTRY-LEVEL FIXED EFFECTS IN PLACE OF COUNTRY-LEVEL RANDOM EFFECTS.**

| Variable | Model specification | |
| --- | --- | --- |
|  | 5 (from Table 3) | 5a |
| *Intercept, by country*** |  |  |
| Benin | 9.19 (0.13) | 9.28 (0.13) |
| Ghana | 9.41 (0.10) | 9.35 (0.10) |
| Honduras | 9.87 (0.09) | 9.87 (0.10) |
| Moldova | 9.75 (0.12) | 9.77 (0.15) |
| Uganda | 8.71 (0.12) | 8.71 (0.14) |
| Zambia | 9.72 (0.10) | 9.75 (0.13) |
| *Coefficient on log(doses), by country*** |  |  |
| Benin | 1.04 (0.11) | 0.98 (0.12) |
| Ghana | 0.63 (0.08) | 0.62 (0.08) |
| Honduras | 1.16 (0.05) | 1.16 (0.05) |
| Moldova | 1.55 (0.08) | 1.59 (0.09) |
| Uganda | 1.14 (0.08) | 1.14 (0.08) |
| Zambia | 0.83 (0.09) | 0.75 (0.09) |
| log(doses) sq | 0.10 (0.03) | 0.11 (0.03) |
| *Other predictors* |  |  |
| log(GDP) | 0.39 (0.14) | --- |
| Govt owned | -0.15 (0.08) | -0.16 (0.08) |
| Hospital | 0.27 (0.08) | 0.28 (0.08) |
| Percent outreach | 0.08 (0.03) | 0.09 (0.03) |
| Percent mgmt | 0.13 (0.03) | 0.13 (0.03) |
| DTP3 per dose | 0.13 (0.02) | 0.13 (0.02) |
| Rural | -0.04 (0.06) | -0.05 (0.06) |
| ANC4 | 0.12 (0.07) | 0.13 (0.06) |
| Wealth ratio | -0.07 (0.03) | -0.08 (0.03) |
| *Variance parameters* |  |  |
| Error term | 0.32 (0.01) | 0.32 (0.01) |
| SD of country r.e.s, intercept | 0.24 (0.21) | --- |
| SD of province r.e.s, intercept | 0.14 (0.03) | 0.14 (0.03) |
| SD of country r.e.s, log(doses) | 0.46 (0.23) | --- |
| WAIC | 212.4 | 212.7 |
| Sample size | 316 | 316 |

* Model 5a is similar to Model 5 except country fixed effects are used in place of random effects (applies to the intercept and the coefficient on log(doses). Log(GDP) is also omitted from Model 5a as a coefficient for this predictor cannot be estimated with country fixed effects.

** For random effects specification (Model 5), country intercepts in the table calculated as the overall intercept plus the country random-effect for the intercept, plus the coefficient on log(GDP) multiplied by log(GDP) for each country. Coefficient on log(doses) calculated as the overall coefficient plus the country-level random effect for this predictor. These calculations undertaken to make reported values directly comparable between models.

**TABLE S6: SENSITIVITY ANALYSIS FOR REGRESSION OF LOG TOTAL COST ON DOSES AND OTHER PREDICTORS: INCLUSION OF DISTRICT-LEVEL RANDOM EFFECTS.**

| Variable* | Model specification | |
| --- | --- | --- |
|  | 5 (from Table 3) | 5b* |
| Intercept | 9.48 (0.13) | 9.47 (0.13) |
| *Service volume* |  |  |
| log(doses) | 1.04 (0.20) | 1.06 (0.21) |
| log(doses) sq | 0.10 (0.03) | 0.09 (0.03) |
| *Other predictors* |  |  |
| log(GDP) | 0.39 (0.14) | 0.37 (0.15) |
| Govt owned | -0.15 (0.08) | -0.12 (0.08) |
| Hospital | 0.27 (0.08) | 0.27 (0.08) |
| Percent outreach | 0.08 (0.03) | 0.06 (0.03) |
| Percent mgmt | 0.13 (0.03) | 0.13 (0.03) |
| DTP3 per dose | 0.13 (0.02) | 0.12 (0.02) |
| Rural | -0.04 (0.06) | -0.05 (0.06) |
| ANC4 | 0.12 (0.07) | 0.13 (0.07) |
| Wealth ratio | -0.07 (0.03) | -0.07 (0.03) |
| *Random effects included* |  |  |
| Country r.e.s for intercept | + | + |
| Province r.e.s for intercept | + | + |
| District r.e.s for intercept | – | + |
| Country r.e.s for log(doses) | + | + |
| *Variance parameters* |  |  |
| Error term | 0.32 (0.01) | 0.30 (0.01) |
| SD of country r.e.s, intercept | 0.24 (0.21) | 0.22 (0.20) |
| SD of province r.e.s, intercept | 0.14 (0.03) | 0.10 (0.04) |
| SD of district r.e.s, intercept | --- | 0.15 (0.03) |
| SD of country r.e.s, log(**doses**) | 0.46 (0.23) | 0.46 (0.23) |
| WAIC | 212.4 | 192.8 |
| Sample size | 316 | 316 |

* Model 5b is similar to Model 5 with the addition of district random effects for the intercept.

**TABLE S7: SENSITIVITY ANALYSIS FOR REGRESSION OF LOG TOTAL COST ON DOSES AND OTHER PREDICTORS: ROBUST REGRESSION WITH ERRORS DISTRIBUTED STUDENT T.**

| Variable* | Model specification | |
| --- | --- | --- |
|  | 5 (from Table 3) | 5c* |
| Intercept | 9.48 (0.13) | 9.47 (0.17) |
| *Service volume* |  |  |
| log(doses) | 1.04 (0.20) | 1.06 (0.22) |
| log(doses) sq | 0.10 (0.03) | 0.10 (0.03) |
| *Other predictors* |  |  |
| log(GDP) | 0.39 (0.14) | 0.38 (0.16) |
| Govt owned | -0.15 (0.08) | -0.16 (0.08) |
| Hospital | 0.27 (0.08) | 0.29 (0.09) |
| Percent outreach | 0.08 (0.03) | 0.09 (0.03) |
| Percent mgmt | 0.13 (0.03) | 0.14 (0.03) |
| DTP3 per dose | 0.13 (0.02) | 0.13 (0.02) |
| Rural | -0.04 (0.06) | -0.03 (0.06) |
| ANC4 | 0.12 (0.07) | 0.11 (0.07) |
| Wealth ratio | -0.07 (0.03) | -0.07 (0.03) |
| *Random effects included* |  |  |
| Country r.e.s for intercept | + | + |
| Province r.e.s for intercept | + | + |
| Country r.e.s for log(doses) | + | + |
| *Variance parameters* |  |  |
| Error term | 0.32 (0.01) | 0.29 (0.02) |
| d.f. for error term | --- | 17.2 (10.9) |
| SD of country r.e.s, intercept | 0.24 (0.21) | 0.27 (0.24) |
| SD of province r.e.s, intercept | 0.14 (0.03) | 0.15 (0.03) |
| SD of country r.e.s, log(doses) | 0.46 (0.23) | 0.46 (0.22) |
| WAIC | 212.4 | 210.5 |
| Sample size | 316 | 316 |

* Model 5c is similar to Model 5 except with a robust regression approach to allow for outliers. Errors are assumed to follow a Student’s *t* distribution. A weakly-informative Gamma(2, 0.1) prior was used for the degrees of freedom parameter.

**TABLE S8: SENSITIVITY ANALYSIS FOR REGRESSION OF LOG TOTAL COST ON DOSES AND OTHER PREDICTORS: NON-INFORMATIVE PRIORS USED FOR REGRESSION COEFFICIENTS AND VARIANCE TERMS.**

| Variable* | Model specification | |
| --- | --- | --- |
|  | 5 (from Table 3) | 5d* |
| Intercept | 9.48 (0.13) | 9.48 (0.12) |
| *Service volume* |  |  |
| log(doses) | 1.04 (0.20) | 1.05 (0.21) |
| log(doses) sq | 0.10 (0.03) | 0.10 (0.03) |
| *Other predictors* |  |  |
| log(GDP) | 0.39 (0.14) | 0.38 (0.13) |
| Govt owned | -0.15 (0.08) | -0.15 (0.08) |
| Hospital | 0.27 (0.08) | 0.27 (0.08) |
| Percent outreach | 0.08 (0.03) | 0.08 (0.03) |
| Percent mgmt | 0.13 (0.03) | 0.13 (0.03) |
| DTP3 per dose | 0.13 (0.02) | 0.13 (0.02) |
| Rural | -0.04 (0.06) | -0.04 (0.06) |
| ANC4 | 0.12 (0.07) | 0.12 (0.07) |
| Wealth ratio | -0.07 (0.03) | -0.07 (0.03) |
| *Random effects included* |  |  |
| Country r.e.s for intercept | + | + |
| Province r.e.s for intercept | + | + |
| Country r.e.s for log(doses) | + | + |
| *Variance parameters* |  |  |
| Error term | 0.32 (0.01) | 0.32 (0.01) |
| SD of country r.e.s, intercept | 0.24 (0.21) | 0.24 (0.19) |
| SD of province r.e.s, intercept | 0.14 (0.03) | 0.14 (0.03) |
| SD of country r.e.s, log(doses) | 0.46 (0.23) | 0.46 (0.23) |
| WAIC | 212.4 | 212.0 |
| Sample size | 316 | 316 |

* Model 5d is similar to Model 5 except with non-informative priors (improper Uniform distributions) adopted for regression coefficients and variance terms.**TABLE S9: RESULTS FOR REGRESSIONS USING TOTAL DTP3 AS MEASURE OF SERVICE DELIVERY VOLUME*.**

| Variable* | Model specification | | | | |
| --- | --- | --- | --- | --- | --- |
|  | 11 | 12 | 13 | 14 | 15 |
| Intercept | 9.44 (0.45) | 9.42 (0.28) | 9.43 (0.16) | 9.43 (0.17) | 9.49 (0.13) |
| *Service volume* |  |  |  |  |  |
| log(DTP3) | --- | 1.11 (0.03) | 1.10 (0.03) | 1.12 (0.04) | 1.02 (0.30) |
| log(DTP3) sq | --- | -0.02 (0.02) | -0.01 (0.02) | -0.01 (0.02) | 0.12 (0.03) |
| *Other predictors* |  |  |  |  |  |
| log(GDP) | --- | --- | 0.42 (0.16) | 0.33 (0.18) | 0.39 (0.14) |
| Govt owned | --- | --- | -0.12 (0.09) | -0.11 (0.09) | -0.13 (0.09) |
| Hospital | --- | --- | 0.37 (0.09) | 0.35 (0.09) | 0.34 (0.09) |
| Percent outreach | --- | --- | 0.10 (0.04) | 0.10 (0.04) | 0.07 (0.03) |
| Percent mgmt | --- | --- | 0.14 (0.03) | 0.13 (0.03) | 0.12 (0.03) |
| DTP3 per dose |  |  | -0.56 (0.03) | -0.06 (0.03) | -0.04 (0.02) |
| Rural | --- | --- | --- | 0.00 (0.07) | -0.06 (0.06) |
| ANC4 | --- | --- | --- | 0.12 (0.09) | 0.10 (0.07) |
| Wealth ratio | --- | --- | --- | -0.05 (0.04) | -0.06 (0.03) |
| *Random effects included* |  |  |  |  |  |
| Country r.e.s for intercept | + | + | + | + | + |
| province r.e.s for intercept | + | + | + | + | + |
| Country r.e.s for log(doses) | – | – | – | – | + |
| *Variance parameters* |  |  |  |  |  |
| Error term | 0.86 (0.04) | 0.40 (0.02) | 0.37 (0.02) | 0.37 (0.02) | 0.34 (0.01) |
| SD of country r.e.s, intercept | 0.93 (0.48) | 0.60 (0.29) | 0.34 (0.21) | 0.33 (0.25) | 0.25 (0.19) |
| SD of province r.e.s, intercept | 0.44 (0.10) | 0.19 (0.04) | 0.20 (0.04) | 0.20 (0.04) | 0.15 (0.03) |
| SD of country r.e.s, log(doses) | --- | --- | --- | --- | 0.51 (0.37) |
| WAIC** | 828.2 | 348.2 | 304.9 | 305.5 | 258.2 |

*** Models 11-15 match Models 1-5 shown in Table 3, except with doses replaced by DTP3.**

FIGURE S3: TOTAL AND AVERAGE COST CURVES FOR TOTAL DTP3, CALCULATED FROM THE RESULTS OF MODEL 15.

**TABLE S10: SENSITIVITY ANALYSIS FOR REGRESSION OF LOG TOTAL COST ON DOSES AND OTHER PREDICTORS: DIFFERENT FORMULATIONS OF DTP3 COVERAGE.**

| Variable* | Model specification | |  | |
| --- | --- | --- | --- | --- |
|  | 21 | 22^†^ | | 23^‡^ |
| Intercept | 9.47 (0.15) | 9.47 (0.11) | | 9.22 (0.87) |
| *Service volume* |  |  | |  |
| log(doses) | 1.11 (0.21) | 1.07 (0.22) | | 0.60 (0.14) |
| log(doses) sq | 0.08 (0.03) | 0.09 (0.03) | | 0.05 (0.02) |
| *Other predictors* |  |  | |  |
| log(GDP) | 0.43 (0.15) | 0.40 (0.13) | | -0.16 (0.79) |
| Govt owned | -0.16 (0.08) | -0.15 (0.08) | | -0.18 (0.08) |
| Hospital | 0.27 (0.08) | 0.27 (0.08) | | 0.34 (0.09) |
| Percent outreach | 0.09 (0.03) | 0.08 (0.03) | | 0.09 (0.02) |
| Percent mgmt | 0.13 (0.03) | 0.13 (0.03) | | 0.08 (0.02) |
| DTP3 per dose | 0.14 (0.02) | 0.13 (0.02) | | 0.13 (0.02) |
| Rural | -0.01 (0.06) | -0.03 (0.06) | | 0.04 (0.06) |
| ANC4 | 0.11 (0.07) | 0.11 (0.07) | | 0.04 (0.03) |
| Wealth ratio | -0.08 (0.03) | -0.08 (0.03) | | -0.10 (0.03) |
| DTP3 coverage (top-coded) | -0.07 (0.02) | --- | | --- |
| DTP3 coverage (estimated) | --- | -0.05 (0.04) | | --- |
| log(population) | --- | --- | | 0.14 (0.05) |
| *Random effects included* |  |  | |  |
| Country r.e.s for intercept | + | + | | + |
| Province r.e.s for intercept | + | + | | + |
| Country r.e.s for log(doses) | + | + | | + |
| *Variance parameters* |  |  | |  |
| Error term | 0.31 (0.01) | 0.31 (0.01) | | 0.32 (0.02) |
| SD of country r.e.s, intercept | 0.27 (0.21) | 0.22 (0.18) | | 1.63 (1.08) |
| SD of province r.e.s, intercept | 0.15 (0.03) | 0.14 (0.03) | | 0.16 (0.04) |
| SD of country r.e.s, log(**doses**) | 0.42 (0.22) | 0.46 (0.24) | | 0.26 (0.18) |
| WAIC | 202.3 | 211.7 | | 167.9 |
| Sample size | 316 | 316 | | 245 |

*** Model 21 is similar to Models 5 with the addition of the reported DTP3 coverage variable, top-coded to 100%.**

^†^ **Model 22 is similar to Models 5 with the addition of estimated DTP3 coverage, where this variable has been calculated using a formal measurement error model (described below).**

^‡^ **Model 30 is similar to Models 5 except with the addition of the log of reported catchment population (log(population)). This model was fit to a subset of the data (values not available for Honduras) and so WAIC values are not comparable with other models.**

## MEASUREMENT ERROR MODEL FOR DTP3 COVERAGE (MODEL 22)

Many of the reported values of DTP3 coverage were greater than 100%. We interpreted this as estimation error in the size of the target population, which are sometimes based on census estimates that are many years old. Figure S4 shows the distribution of reported DTP3 coverage estimates.

**FIGURE S4: HISTOGRAM OF REPORTED DTP3 COVERAGE*.**


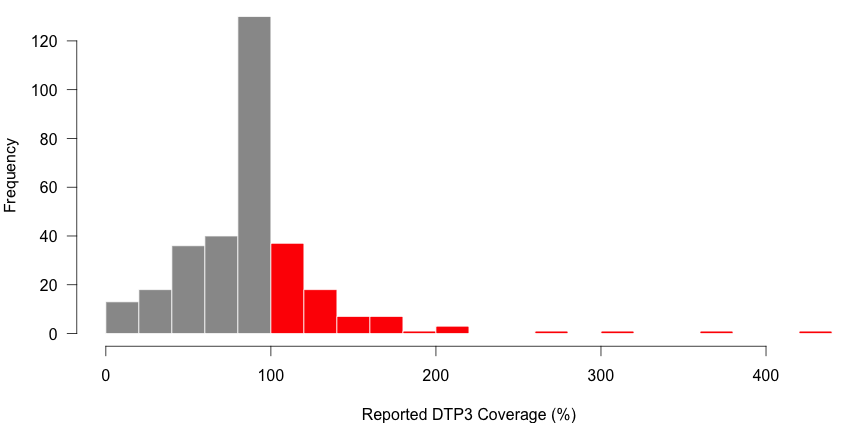


*** Values shown in red represent illogical values (coverage >100%).**

As a sensitivity analysis we constructed a measurement error model that allowed for mismeasurement of the denominator of the DTP3 coverage calculation. **In this model, reported estimates of the DTP3 target population (infants <1 year of age) were assumed to be an imprecise estimate of the ‘true’ target population size. These two values were related with the following equation:**

$$ln\left( {Target\_pop\_true}_{i} \right)=\gamma_{0c}+\gamma_{1c}*ln\left( {Target \_pop\_reported}_{i} \right)+\varepsilon_{i}$$

If reported values for the target population size ($Target \_pop\_reported$) were unbiased on expectation,$\gamma_{0c}$ would be equal to 0.0 and $\gamma_{{1c}_{i}}$ would be equal to 1.0 for each country. If the estimated target population were both unbiased and precise, $\gamma_{0c}$ would be equal to 0.0, $\gamma_{{1c}_{i}}$ would be equal to 1.0, and $\varepsilon_{i}$would be zero.

We fitted these regression equations for each country with informative priors for the coefficients (Normal(0,1) for $\gamma_{0c}$ and Normal(1,1) for $\gamma_{1c}$) and for the error term (HalfCauchy(0,1)). We also restricted the value of $Target pop\_true$ **to be equal to or greater than reported DTP3 volume for each facility, such that DTP3 coverage calculated using** $Target pop\_true$ **was restricted to the range 0-100%**. In this model the ‘true’ value of DTP3 coverage for each site is treated as a random variable, calculated by dividing reported DTP3 volume by the ‘true’ target population size (${Pop\_true}_{i}$as defined above). These ‘true’ DTP3 coverage values were estimated simultaneously with other model parameters, and used in place of the original DTP3 coverage variable as a RHS variable in Model 22 (Table S10). To help identify the model we also added Normal priors for the average DTP3 coverage achieved within each country, centered at reported national coverage in 2011 +/- 1%. Table S11 shows these national values along with estimated sample average values, as well as coefficient values for the imputation model. Figure S5 compares reported and estimated DTP3 coverage values.

**TABLE S11: NATIONAL DTP3 COVERAGE ESTIMATES AND VALUES ESTIMATED BY MEASUREMENT ERROR MODEL (MODEL 22).**

| Country | National DTP3 coverage[2] | Average sample DTP3 coverage (Model 22) | Regression coefficients for measurement error model | | |
| --- | --- | --- | --- | --- | --- |
|  |  |  | $\gamma_{0c}$ | $\gamma_{1c}$ | $\sigma_{c}$ |
| Benin | 75% | 75% (74, 76) | 0.95 (0.52) | 0.82 (0.08) | 0.23 (0.05) |
| Ghana | 91% | 90% (89, 90) | 0.82 (0.35) | 0.87 (0.06) | 0.32 (0.06) |
| Honduras | 91% | 89% (88, 90) | 0.08 (0.10) | 0.98 (0.02) | 0.15 (0.03) |
| Moldova | 93% | 91% (91, 92) | -0.06 (0.14) | 0.99 (0.04) | 0.28 (0.05) |
| Uganda | 82% | 81% (80, 82) | 1.27 (0.58) | 0.85 (0.10) | 0.61 (0.10) |
| Zambia | 81% | 80% (79, 81) | 0.29 (0.41) | 0.93 (0.06) | 0.25 (0.05) |

**FIGURE S5: REPORTED VS. ESTIMATED VALUES OF DTP3 COVERAGE FROM MEASUREMENT ERROR MODEL (MODEL 22).**

# Citations

1. World Bank: **World Development Indicators: GDP per capita (**<http://data.worldbank.org/indicator/NY.GDP.PCAP.CD>**)**. In*.*: World Bank; 2016.

2. WHO: **WHO Global Health Observatory Data Repository (**<http://apps.who.int/gho/data/view.main.80200>**).** In*.* Geneva: WHO; 2016.

3. UNICEF: **Immunization Summary: A statistical reference containing data through 2011 (**<http://www.unicef.org/immunization/files/EN-ImmSumm-2013.pdf>**).** In*.* New York: UNICEF; 2013.

4. Gelman A: **Prior distributions for variance parameters in hierarchical models**. *Bayesian Analysis* 2006, **1**(3):515-533.

5. Winship C, Radbill L: **Sampling Weights and Regression Analysis**. *Sociological Methods Research* 1994, **23**(2):230-257.

6. Gelman A, Hwang J, Vehtari A: **Understanding predictive information criteria for Bayesian models**. *Statistics and Computing* 2014, **24**:997-1016.

7. Vehtari A, Gelman A: **WAIC and cross-validation in Stan**. In*.* Finland.: Aalto University; 2014.

8. Hoffman MD, Gelman A: **The No-U-Turn sampler: adaptively setting path lengths in Hamiltonian Monte Carlo**. *J Mach Learn Res* 2014, **15**

(1):1593-1623.

9. Carpenter B, Gelman A, Hoffman MD, Lee D, Goodrich B, Betancourt M, Brubaker M, Guo J, Li P, Riddell A: **Stan: A probabilistic programming language.** *Journal of Statistical Software* 2017, **76**(1).

10. Stan Development Team: **RStan: the R interface to Stan. R package version 2.15.1 (**<http://mc-stan.org/>**). 2017**. In*.*

11. R Core Team: **R: A language and environment for statistical computing**. In*.* Vienna, Austria: R Foundation for Statistical Computing; 2016.
